# A capture methyl-seq protocol with improved efficiency and cost-effectiveness using pre-pooling and enzymatic conversion

**DOI:** 10.1186/s13104-023-06401-3

**Published:** 2023-07-06

**Authors:** Keita Hasegawa, Kazuhiko Nakabayashi, Keisuke Ishiwata, Yoshifumi Kasuga, Kenichiro Hata, Mamoru Tanaka

**Affiliations:** 1 Department of Maternal-Fetal Biology, National Center for Child Health and Development, 2-10-1 Okura, Setagaya-ku, Tokyo, 157-8535 Japan; 2 Department of Obstetrics and Gynecology, Keio University School of Medicine, Shinjuku, Tokyo, 160-0016 Japan; 3 Department of Human Molecular Genetics, Gunma University Graduate School of Medicine, Maebashi, Gunma 371-8511 Japan

**Keywords:** DNA methylation, Capture methyl-seq, Enzymatic conversion, TET2, APOBEC

## Abstract

**Objective:**

The opportunities for sequencing-based methylome analysis of clinical samples are increasing. To reduce its cost and the amount of genomic DNA required for library preparation, we aimed to establish a capture methyl-seq protocol, which adopts pre-pooling of multiple libraries before hybridization capture and TET2/APOBEC-mediated conversion of unmethylated cytosine to thymine.

**Results:**

We compared a publicly available dataset generated by the standard Agilent protocol of SureSelect XT Human Methyl-Seq Kit and our dataset obtained by our modified protocol, EMCap, that adopted sample pre-pooling and enzymatic conversion. We confirmed that the quality of DNA methylation data was comparable between the two datasets. As our protocol, EMCap, is more cost-effective and reduces the amount of input genomic DNA, it would serve as a better choice for clinical methylome sequencing.

**Supplementary Information:**

The online version contains supplementary material available at 10.1186/s13104-023-06401-3.

## Introduction

DNA methylation is one of the well-studied epigenetic systems in mammals [1], and alteration of DNA methylation at specific regions, such as gene promoters, has been reported to be associated with disease development [1, 2]. DNA methylation has been examined extensively through multi-institutional consortium efforts such as The Cancer Genome Atlas (TCGA) to elucidate cancer-associated alterations of DNA methylation [3] and to identify the association between the variation in DNA methylation and the common diseases/phenotypes in many epigenome-wide association studies (EWAS) [4]. Recent studies have also demonstrated the utility of DNA methylation information to diagnose rare monogenic disorders [5]. As such, the opportunities for obtaining DNA methylation profiles of clinical samples are expected to further increase. Although it is technically possible to obtain DNA methylation information in a genome-wide manner by the methods such as EM-seq [6] and long-read HiFi sequencing [7], it is currently not practical cost-wise to apply such methods to hundreds to thousands of samples. Targeted capture methylation sequencing (methyl-seq) significantly reduces per-sample costs and is expected to remain as one of the methods for clinical methylome analyses for the time being. A few biotechnology companies offer custom bait design with a broad scalability for target region sizes, and Agilent Technologies is one of them. The current Agilent’s SureSelect XT Human Methyl-Seq protocol adopts bisulfite conversion and is not compatible with pre-pooling of genomic DNA libraries prior to hybridization, which makes it difficult to apply this platform to samples with a small amount of DNA as well as to save the cost for capture reagents by pooling pre-capture libraries. In this study, we aimed to establish a capture methyl-seq protocol that adopts pre-pooling of genomic DNA libraries and uses enzymatic conversion to detect methylated cytosines.

## Main text

### Materials and methods

All methods were carried out in accordance with relevant guidelines and regulations under Ethics approval and consent to participate.

#### Preparation of DNA

Genomic DNA (gDNA) samples (n = 4) were obtained from the BioBank at the National Center for Child Health and Development. gDNA was extracted from whole blood using the MagCore Genomic DNA Large Volume Whole Blood Kit (RBC Bioscience, MGB1200). DNA samples (1000 ng) dissolved in 80 μL of 0.1X TE buffer (pH 8.0) were sheared with Covaris S220 system (Covaris) to the peak-top size of 300 bp using the following conditions: 140W of peak incident power, 10% of duty factor, 200 cycles per burst, and 90 s of treatment time.

#### End repair, dA-tailing, and adapter ligation

All enzymatic reaction mixtures described hereafter were prepared on ice and mixed by pipetting unless otherwise specified. All isothermal incubation and thermal cycling processes were conducted on a ProFlex 3 × 32-well PCR System (Thermo Fisher Scientific) using PCR eight-tube strips (0.2 mL) and eight-cap strips. Fragmented DNA (200 ng in 16 µL) was filled up to 43 µL with water (Nacalai tesque, 06442-95) and added to 7 µL of NEBNext Ultra II End Prep Reaction Buffer and 3 µL of End Prep Enzyme Mix (NEB, E7120). The mixture was incubated at 20 °C for 30 min and at 65 °C for 30 min (the heated lid was set to 75 °C). Subsequently, 2.5 µL of an xGen Methyl UDI-UMI adapter (15 µM; IDT, 10006644; Additional file 1: Text S1), 1 µL of NEBNext Ultra II Ligation Enhancer and 30 µL of Ligation Master Mix (NEB, E7120) were added to the reaction mixture. The mixture (93.5 µL in total) was incubated at 20 °C for 60 min (heated lid off). After the ligation reaction, the reaction mixture was purified using 110 µL of NEBNext Sample Purification Beads (NEB, E7120) according to the manufacturer’s instruction (see the Additional file 1: Text Additional file: 2: Text S2). The adapter-ligated DNA was eluted with 28 µL of Elution Buffer (NEB, E7120).

#### Oxidation of 5-methylcytosines and 5-hydroxymethylcytosines by Tet methylcytosine dioxygenase (TET2)

Ten µL of TET2 Reaction Buffer containing TET2 Reaction Buffer Supplement, 1 μL of Oxidation Supplement, 1 μL of DTT, 1 μL of Oxidation Enhancer (T4-phage beta-glucosyltransferase (T4-BGT)) and 4 μL of TET2 (NEB, E7120) were added to the 28 μL of adapter-ligated DNA (45 µL in total). While the DNA mixture was kept on ice, 1 µL of Fe (II) solution (500 mM, NEB, E7120) was diluted to 4 µM by adding 1249 μl of water. Five μL of the diluted Fe (II) solution was added to the DNA mixture. The mixture (50 µL in total) was incubated at 37 °C for 60 min (the heated lid set to 45 °C). After 1 µL of Stop Reagent (NEB, E7120) was added, the mixture was further incubated at 37 °C for 30 min. The reaction mixture was purified using 90 µL of NEBNext Sample Purification Beads (see the Additional file 1: Text S1). The TET2-treaded DNA was eluted with 12 µL of water.

#### Sample pooling and hybridization

Up to 4 samples of the adapter-ligated and TET2-treated DNA (12 µL each) were pooled. After 5 µL of Human Cot DNA (IDT, 1080577) was added to the pool, the pooled DNA was dried up using a centrifugal concentrator (CC-105, TOMY Digital Biology) for 45 min, and resuspended in 12 µL of water. After 1.25 µL of xGen Universal Blockers TS Mix (IDT, 1075474) was added, the DNA solution (13.25 µL in total) was subjected to the follow three incubation steps: 95 °C for 5 min, 65 °C for 10 min, and 65 °C for 1 min (heated lid set to 105 °C). During the first incubation step, 2 µL of 25% SureSelect RNase Block (Agilent Technologies, 5190-9686) was prepared and kept on ice. During the second step, a probe hybridization mixture was prepared by mixing 2 µL of 25% of SureSelect RNAse Block, 6 µL of SureSelect Fast Hybridization Buffer (Agilent Technologies, 5190-9686), and 5 µL of SureSelect XT Human Methyl-Seq Capture Library (biotin-labelled RNA baits) (Agilent Technologies, 5190-4661), and kept at room temperature. During the third step, the probe hybridization mixture (13 µL in total) was added to the DNA solution (13.25 µL) on a thermal cycler block. Subsequently, the mixture (26.25 µL in total) was incubated under the following conditions, sixty cycles of 65 °C for 1 min and 37 °C for 3 s, for the hybridization of RNA baits to the adapter-ligated and TET2-treated DNA.

#### Capture and wash of DNA/RNA hybrids

The capture and wash of DNA/RNA hybrids were conducted using Dynabeads MyOne Streptavidin T1 magnetic beads (Thermo Fisher Scientific, 65601) and binding/wash buffers in the SureSelect XT HS and XT Low Input kits (Agilent Technologies, 5190-9687) according to the manufacturer’s protocol. Briefly, the hybridized DNA solution was added to the streptavidin beads resuspended in the SureSelect Bindig Buffer and mixed by a plate mixer for 30 min at room temperature. The beads were washed with SureSelect Wash Buffer 1 once at room temperature and subsequently six times with SureSelect Wash Buffer 2 prewarmed at 70 °C. The detailed procedures were described in the Additional file 1: Text S1.

#### Elution of DNA and deamination of cytosines by apolipoprotein B mRNA editing enzyme catalytic subunit (APOBEC)

After completely removing the residual buffer at the final wash procedure, the 0.2 mL PCR tube containing the streptavidin beads was removed from the magnetic stand. Subsequently, 4 µL of 0.1N NaOH was added to the tube. The tube was centrifuged briefly for spin-down, vortexed for mixing, and centrifuged briefly again. It is important to resuspend the streptavidin beads with 0.1N NaOH by using a vortex mixer. When the beads were mixed by pipetting, the beads tended to remain inside the tip and could not be recovered, which led to a lower yield of the final libraries. After the tube was placed on a magnetic stand for 1 min, and the supernatant (4 µL), which is expected to contain denatured single-stranded DNA, was transferred to a new PCR tube. Eighty-four µL of water, 10 µL of APOBEC Reaction Buffer, 1 µL of bovine serum albumin (BSA) and 1 µL of APOBEC (NEB, E7120) were added to 4 µL of eluted single-stranded DNA. The mixture (100 µL in total) was incubated at 37 °C for 3 h (the heated lid set to 45 °C). After this APOBEC reaction, the mixture was purified using 100 µL of NEBNext Sample Purification Beads (see the Additional file 1: Text S1, Additional file 2: Text S2) and eluted with 20 µL of Elution Buffer (NEB, E7120). The eluted DNA was transferred to a new 0.2 mL PCR tube.

#### PCR amplification and quantification of post-capture library

Quantities of 2.5 µL each of PCR primers (10 µM; 5′-AATGATACGGCGACCACCGA-3′ [P5] and 5′-CAAGCAGAAGACGGCATACGA-3′ [P7]) and 25 µL of NEBNext Q5U Master Mix (NEB, E7120) were added to the eluted DNA (50 µL in total). The mixture was subjected to the following thermal cycling conditions: 98 °C for 30 s, then nine cycles of 98 °C for 10 s, 62 °C for 30 s, and 65 °C for 60 s, and finally 65 °C for 5 min. After PCR amplification, the mixture was purified using 45 µL of NEBNext Sample Purification Beads and eluted with 21 µL of Elution Buffer (NEB, E7120). The eluted DNA was transferred to a new tube. The size distribution and the concentration of the amplified capture library were examined using the High Sensitivity Kit (Agilent, 5067-4626) and 2100 Bioanalyzer. The entire protocol for the preparation of EMCap libraries is provided as Additional file 2: Text S2.

#### Illumina sequencing

The final libraries were subjected to library quantification and paired-end sequencing (151 bp × 2) using HiSeq X Ten Reagent Kit v2.5 (FC501-2521) on a HiSeq-X system (Illumina) at Macrogen Japan Corp. (Tokyo, Japan) with a 20% amount of spike-in PhiX Control v3 Library (Illumina) for colour balancing of low nucleotide diversity. The BCL (base calls) files were converted to fastq files using the bcl2fastq software (Illumina). The sequencing data were analyzed as described in the Additional file 1: Text S1.

### Results and discussion

**Fig. 1:**
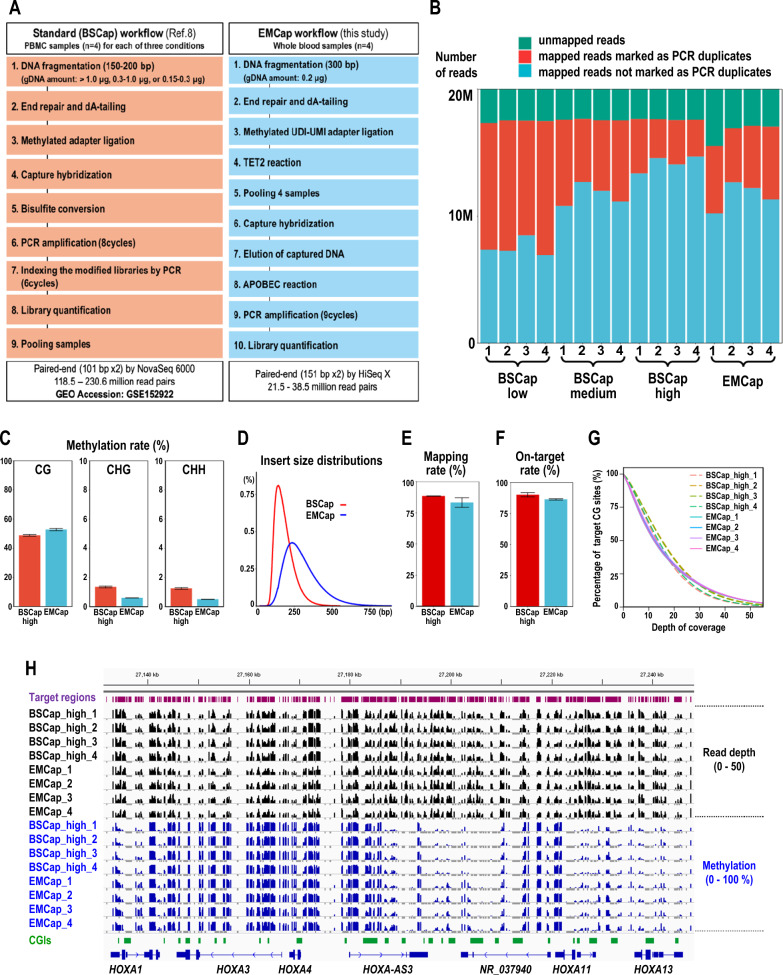
Comparisons of experimental procedures and sequencing metrics between the Agilent’s SureSelect XT Human Methyl-Seq (BSCap) and EMCap. **A** Comparison of experimental procedures. **B** Comparison of PCR duplication rates between the BSCap data (GSE152922) and the EMCap data. “High”, “Mid”, and “Low” correspond to the amounts of genomic DNA used for BSCap library preparation, i.e., > 1000 ng, 300–1000 ng, and 150–300 ng, respectively. **C**–**H**. Comparison between BSCap_high and EMCap libraries. **C** Mean methylation rates of cytosines in CpG, CHG, and CHH nucleotides. **D** Insert size distributions. **E** Mapping rates. **F** On-target rates. **G** Cumulative plots of the read depth for the 3,147,687 target CpG sites covered in one or more libraries (read depth >  = 1) for each of four EMCap and four BSCap_high libraries. **H** Read depths and methylation values of individual target CpG sites with the minimum coverage of 10 or greater among eight libraries visualized for a 120 kb interval (hg19) from the *HOXA* gene cluster region on chromosome 7 using Integrative Genomics Viewer (IGV; https://igv.org/). An igv format file for data visualization was generated as described previously [11]. Genomic intervals of the capture targets are shown in the “Target regions” track by visualizing the bait information (S03770311_Covered.bed) for the SureSelect XT Human Methyl-Seq Capture Library (Agilent). The “CGIs” track shows the positions of the CpG islands retrieved from the UCSC Genome Browser (https://genome.ucsc.edu/). Only a subset of genes within the interval is shown for simplicity. Negative values were assigned (− 10 for read depth and − 1 for methylation) when the read depth for a target CpG site is zero, and are shown in gray bars to help distinguishing “missing value” from “0% methylation”. The data ranges of non-negative values are 0 to 50 for read depth and 0 to 1 (0 to 100%) for DNA methylation

We modified the Agilent’s SureSelect XT Human MethylSeq protocol by replacing the bisulfite conversion procedure to the enzymatic conversion and by adopting unique dual-indexed (UDI) methylated adapters. To distinguish Agilent’s and our capture methyl-seq protocols, we hereafter refer to these protocols as “BSCap” and “EMCap” based on their DNA conversion methods, that is, bisulfite (BS) conversion and the NEBNext Enzymatic Methyl-seq (EM) Conversion Module (Fig. 1A). TET2 and APOBEC reactions were integrated in the EMCap workflow as the pre-capture and the post capture procedures, respectively (Fig. 1A).

We prepared capture methyl-seq libraries for four peripheral blood DNA samples by the EMCap protocol using the capture baits of the SureSelect XT Human Methyl-Seq Kit, and obtained sequencing data for the capture libraries as described in the Additional file 2: Text S1 (also see Additional file 9: Table S1). The capture baits target 3,156,463 CG sites covering the majority of gene promoters and CpG islands and a subset of enhancers [8]. For four EMCap libraries, we obtained 100 times the amount of sequences on average relative to the capture bait size (84 Mb) as a sufficient amount of sequences to evaluate their basic metrics. We used a publicly available dataset GSE152922 [9], which includes SureSelect XT Human Methyl-Seq data for four peripheral blood mononuclear cells with three different amounts of input DNA (high, > 1000 ng; medium, 300–1000 ng; low, 150–300 ng) as BSCap data (twelve libraries in total). The BSCap and the EMCap data were analyzed using the same bioinformatic protocols (Additional file 1: Text S1, Additional file 2: Text S2). The quality control check of EMCap was performed by using Bioanalyzer (Additional file 10: Fig. S1A). To compare PCR duplicate rates, 20 million (M) read pairs from each of the libraries were mapped to the hs37d5 reference genome. The average PCR duplicate rates of four types were 12.6% (BSCap_high), 33.6% (BSCap_medium), 56.9% (BSCap_low), and 30.3% (EMCap), demonstrating that the library complexity achieved by the EMCap protocol using 200 ng of input genomic DNA was higher than that by the BSCap_low protocol (two-sample t-test *P* < 0.05) and not significantly different from that by the BSCap_medium protocol (two-sample t-test *P* = 0.34) (Fig. 1B). Because only the BSCap_high fulfilled the manufacturer’s recommendation for the amount of input DNA among three BSCap conditions, BSCap_high and EM_Cap data were further compared (Fig. 1C–H). The comparison results of for conditions (BSCap_low, BSCap_medium, BSCap_high, and EMCap) are shown in the Additional file 10: Fig. S1B–E. In mammalian species, cytosine methylation at CHG and CHH contents is low in most of the differentiated cells including blood cells [10], and therefore can be used to assess the completeness of C to T conversion in methyl-seq data for blood. The methylation rates at CHGs and CHHs were consistently low (< 0.7%) in the EMCap data, suggesting high conversion rates (Fig. 1C). The fragment size distributions determined by mapped positions of read pairs were consistent with the DNA fragmentation conditions of the two protocols (Fig. 1D). The mapping rates (88% and 83%) and on-target rates (90% and 86%) were satisfactorily high in both datasets (Fig. 1E, F). After adjusting mapped base numbers of all eight samples to approximately the same (3776 Mb) by randomly selecting mapped reads from deduplicated bam files, the following metrics were compared between two protocols: the average ratios of CpG sites covered (read depth >  = 1) out of the 3,156,463 target CpG sites were 96.0% and 97.6% for EMCap and BSCap_high libraries, respectively (Additional file 9: Table S2); the average read depths among covered target CpG sites were 16.4 and 16.6 (the Additional file 9: Table S3). The distributions of the read depths for the 3,147,687 CpG sites covered (read depth >  = 1) in at least one of eight libraries were similar between the two protocols (Fig. 1G and the Additional file 10: Fig. S1F). The read depth and methylation data of BSCap_high and EMCap libraries are shown for an approximately 120 kb interval within the *HOXA* gene cluster as an example with two different coverage thresholds: minimum coverage 10 (Fig. 1H) and minimum coverage 1 in at least one of eight libraries (Additional file 10: Fig. S2). These results demonstrate that the overall performance of the EMCap is comparable with that of the BSCap (Fig. 1E–G), and that EMCap is superior to BSCap in efficiently obtaining methylome data from a limited amount of genomic DNA (200 ng) (Fig. 1B). The EMCap protocol also reduces the cost of capture baits, which occupies a major part of the reagent cost, to one-fourth.

### Limitations

There are two limitations in our EMCap protocol. First, the total experimental time of EMCap is longer than that of BSCap (15 h vs 10 h) because of TET2 (1.5 h) and APOBEC (3 h) reactions in the EMCap. Second, when more than four samples were pooled, we obtained sequencing data with lower quality (such as lower mapping and conversion rates), the cause of which is currently unknown. Therefore, when EMCap is conducted, we recommend up to four samples of pre-pooling, and evaluation of mapping and conversion rates by a small-scale sequencing (e.g., using MiSeq) before proceeding to a full-scale sequencing. It should be also noted as a limitation common to EMCap and BSCap that a portion of CpG islands (CGIs) are prone to low coverage likely due to both sequencing and capture biases as observed in some of the CGIs shown in Fig. 1H and Additional file 10: Fig. S2.

## Supplementary Information

**Additional file 1: Text S1.** Supplementary methods.

**Additional file 2: Text S2.** EMCap protocol.

**Additional file 3: Text S3.** Shellscript_A_AdjustReadNumbers.

**Additional file 4: Text S4.** Shellscript_B_Mapping-DNAm-Metrics-Bam.

**Additional file 5: Text S5.** Shellscript_C_InsertSize.

**Additional file 6: Text S6.** Shellscript_D_prep_bam_files_3776Mb.

**Additional file 7: Text S7.** Shellscript_E_bam_to_methylKit.

**Additional file 8: Text S8.** Shellscript_F_Cumulative_ReadDepth.

**Additional file 9: Table S1.** Index information and sequencing stats for four EMCap libraries. **Table S2.** Comparison of the numbers and the ratios of target CpG sites covered (read_depth >= 1) between BSCap_high libraries and EMCap libraries. **Table S3.** Comparison of the average read depth for capture-target CpG sites between BSCap_high libraries and EMCap libraries.

**Additional file 10: Fig. S1.** Comparisons of sequencing metrics among the Agilent’s SureSelect XT Human Methyl-Seq (BSCap) high, medium, low and EMCap. **Fig. S2.** Read depths and methylation values of individual target CpG sites with the minimum coverage of 1 in at least one of the eight libraries visualized for a 120 kb interval (hg19) from the *HOXA* gene cluster region on chromosome 7 using Integrative Genomics Viewer (IGV; https://igv.org/).

## Data Availability

The sequence read files (fastq.gz) were deposited at the Japanese Genotype–phenotype Archive (https://www.ddbj.nig.ac.jp/jga/) (Dataset accession number: JGAD000734). Requests regarding data and materials should be sent to the corresponding author.
