# Supplementary material for: A capture methyl-seq protocol with improved efficiency and cost-effectiveness using pre-pooling and enzymatic conversion
Source: BMC Res Notes. 2023 Jul 6;16:141. doi: 10.1186/s13104-023-06401-3 (PMC10326935; doi:10.1186/s13104-023-06401-3)
Supplement: Supplementary file 1 — Additional file 1: Text S1. Supplementary methods. [file 13104_2023_6401_MOESM1_ESM.docx]

**Additional file Text S1 (Hasegawa K *et al.*)**

**Supplementary Methods**

**Nucleotide sequences of xGen Methyl unique dual index (UDI) unique molecular identifier (UMI) adapters**

The xGen Methyl UDI-UMI Adapters, 1-16 (Integrated DNA Technologies 10006644) have methyl-dC bases instead of regular dC bases​​. Methyl-dC bases are shown as “C” in the following sequences for easier readability​.

p5/i5 strand (5'->3'): Phos/GATCGGAAGAGCGTCGTGTAGGGAAAGAGTGTxxxxxxxxGTGTAGATCTCGGTGGTCGCCGTATCATT​​​; p7/i7 strand (5'->3'): CAAGCAGAAGACGGCATACGAGATNNNNNNNNNxxxxxxxxGTGACTGGAGTTCAGACGTGTGCTCTTCCGATC*T​. “xxxxxxx​x” represents an 8-base index sequence, an “NNNNNNNNN” represents ​9-base unique molecular identifier (UMI) sequences. We adopted these methylated UDI-UMI adapters to our EMCap protocol, primarily because of their methylated and UDI features. We did not use UMI information for the removal of PCR duplicates in this study.

**NEBNext Sample Purification Beads**

A reaction mixture to be purified was added with a designated volume of NEBNext Sample Purification Beads (NEB, E7120), mixed by pipetting, and incubated at room temperature for 5 min. An eight-tube strip (0.2 mL) containing the mixture was placed on a magnetic stand (FastGene, MagnaStand 0.2) for a few minutes until the solution became clear. After the supernatant was removed, the beads were washed with 200 µL of 80% EtOH twice and air-dried for 2 min. The beads were added with a designated amount of Elusion Buffer (NEB, E7120), resuspended, and incubated for 2 min. After the eight-tube strip was placed on the magnetic stand, the supernatant was transferred to a new eight-tube strip.

**Capture and wash of DNA/RNA hybrids**

**i) Preparation of streptavidin beads**

Dynabeads MyOne Streptavidin T1 magnetic beads (Thermo Fisher Scientific, 65601) were washed with SureSelect Binding Buffer (Agilent Technologies, 5190-9687) during the 60 min period before the end of the hybridization procedure as follows. Fifty µL of the beads were added to a 1.5 mL tube containing 200 µL of the binding buffer and mixed well. The tube was placed on a magnetic stand (Thermo Fisher Scientific, DynaMag-2) for 5 min. After the supernatant was removed, the tube was removed from the magnetic stand. The beads were resuspended with 200 µL of the binding buffer. This bead washing procedure was repeated three times. The streptavidin beads resuspended in 200 µL of the binding buffer were transferred to a 0.2 mL tube of a new eight-tube strip.

**ii) Capture of hybridized DNA by streptavidin beads and pre-warming of wash buffer 2**

Immediately after the completion of the hybridization procedure, the hybridized DNA solution (26.25 µL) was transferred to the tube containing the streptavidin beads. The tube was mixed using a MixMate (Eppendorf) at 1600 rpm for 30 min at room temperature. During this mixing procedure, 1400 µL of SureSelect Wash Buffer 2 (Agilent Technologies, 5190-9687) was warmed at 70 °C.

**iii) Wash**

The 0.2 mL tube containing the mixture of the streptavidin beads and hybridized DNA was placed on a magnetic stand for 5 min. After the supernatant was removed, the tube was removed from the stand. The beads were resuspended with 200 µL of SureSelect Wash Buffer 1 (Agilent Technologies, 5190-9687). The tube was placed on a magnetic stand for 5 min, and the supernatant was removed. After the tube was removed from the stand, 200 µL of SureSelect Wash Buffer 2 pre-warmed at 70 °C was added to the beads. After the beads and the buffer were mixed briefly by vortex, the tube was placed on the magnetic stand. The supernatant was removed. This wash procedure using SureSelect Wash Buffer 2 was repeated six times.

**Data analysis**

**i)** Selection of 20 million read pairs from fastq files

To evaluate the basic metrics, such as conversion rates, mapping rates, PCR duplicate rates, and on-target rates, 20 million (M) read pairs were randomly selected from sequencing data (fastq files) of the BSCap and EMCap libraries using **ScriptA_AdjustReadNumbers** (**Additional file 3**).

**ii)** Alignment to the reference genome, DNA methylation analysis, evaluation of library metrics, and generation of deduplicated bam files

Selected or full sequencing data (fastq files) were processed using **ScriptB_Mapping-DNAm-Metrics-Bam** (**Additional file 4**) to obtain DNA methylation data (**Fig.1C**, **Supplementary Fig.1B**), to evaluate mapping rates (**Fig.1BE**, **Supplementary Fig.1C**) and on-target rates (**Fig.1F**, **Supplementary Fig.1D**), to mark PCR duplicates (**Fig.1B**), and to generate bam files with or without PCR duplicates. Using **ScriptB_Mapping-DNAm-Metrics-Bam** (**Additional file 4**) fastq files were processed by TrimGalore! ^1)^ for adapter trimming and for removing low quality reads. Bismark ^2)^ and HISAT2 ^3)^ were used to map sequence reads to the hs37d5 reference genome ^4)^ with “*--non_directional*” option. It should be noted that xGen Methyl UDI-UMI adapters (IDT) as well as xGen UDI-UMI adapters have the insert strandedness flipped (i.e., to the opposite strand) when compared to standard Illumina TruSeq adapters. Cytosine methylation rates for CG, CHG, and CHH sites and mapping rates were obtained from summary output files generated by Bismark. PCR duplicate reads were marked, counted and removed using samtools ^5)^. On-target reads were defined as the number of reads overlapping at least one base pair with a bait region of the SureSelect XT Human Methyl-Seq Capture Library (Agilent). The full list of the 350,538 bait regions, whose total size is 84 Mb, is available as S03770311_Covered.bed at the Agilent’s SureDesign website (https://earray.chem.agilent.com/suredesign/). On-target rate was defined as the ratio of on-target reads to the total number of mapped reads, and was calculated using samtools and bedtools ^6)^.

**iii)** Insert size distributions

The insert size distributions of BSCap and EMCap libraries (**Fig.1D** and **Supplementary Fig.1E**) were determined by analyzing bam files without PCR duplicates using **ScriptC_InsertSize** (**Additional file 5**), and plotted using *R* ^7)^.

**iv)** Read depth analysis of target CG sites (**Fig.1G)** and data visualization (**Fig.1H**)

**ScriptD_prep_bam_files_3776Mb** (**Additional file 6**) was used to generate deduplicated bam files whose mapped base numbers were approximately the same (3,776 Mb) for EMCap and BSCap_high libraries. The resultant bam files were subjected to **ScriptE_bam_to_methylKit** (**Additional file 7**) to process those with *bismark_methylation_extractor* and *coverage2cytosine* commands of Bismark to generate genome-wide cytosine reports (methylKit.txt format). The resultant report files were further processed with MethylKit ^8)^ using *methRead* (mincov=1) and *unite* (destrand=TRUE, min.per.group=1L) commands to obtain a text file containing the read depths and methylation rates of all CG sites for all eight libraries. After a text file containing the read depths for only the on-target CG sites (n=3,147,687) was generated using bedtools ^6)^, the file was processed with **ScriptF_Cumulative_ReadDepth** (**Additional file 8**) to generate cumulative read depth data. The resultant data were plotted using R ^7)^ (**Fig.1G**). The same set of methylKit.txt files were also processed with MethylKit ^8)^ (“mincov=10”) as described previously ^9)^, and converted to an IGV format for data visualization (**Fig.1H**).

**v)** BSCap data

GSE152922 dataset ^10)^ at the Gene Expression Omnibus (GEO) database at NCBI (<https://www.ncbi.nlm.nih.gov/geo/>) was obtained as a dataset generated using the SureSelect XT Human Methyl-Seq Capture Library with the conventional bisulfite conversion method (BSCap). In the corresponding study, human genomic DNA (ranging from 150 ng to 1000 ng or more) extracted from peripheral blood mononuclear cells (PBMC) was sheared to a fragment length of 150-200 bp using the Covaris E220 system. The final capture libraries were subjected to paired-end sequencing (101 bp × 2) on a NovaSeq 6000 (Illumina). SRA files were downloaded, converted to fastq files using the SRA-toolkit ^11)^, and analyzed under the same bioinformatic procedures applied to the EMCap data as described above.

**Supplementary references:**

1. https://www.bioinformatics.babraham.ac.uk/projects/trim_galore/

2. Krueger F, Andrews SR. Bismark: a flexible aligner and methylation caller for Bisulfite-Seq applications. Bioinformatics. 2011;27(11):1571-2.

3. http://daehwankimlab.github.io/hisat2/

4. ftp://ftp.1000genomes.ebi.ac.uk/vol1/ftp/technical/reference/phase2_reference_assembly_sequence/

hs37d5.fa.gz

5. http://www.htslib.org/

6. https://bedtools.readthedocs.io/en/latest/

7. https://www.r-project.org/

8. https://bioconductor.org/packages/release/bioc/vignettes/methylKit/inst/doc/methylKit.html

9. Nakabayashi K, Yamamura M, Haseagawa K, Hata K. Reduced Representation Bisulfite Sequencing (RRBS). Methods Mol Biol. 2023; 2577:39-51.

10. Shu C, Zhang X, Aouizerat BE, Xu K. Comparison of methylation capture sequencing and Infinium MethylationEPIC array in peripheral blood mononuclear cells. Epigenetics Chromatin. 2020; 13(1):51.

11. https://github.com/ncbi/sra-tools
