# Supplementary material for: A capture methyl-seq protocol with improved efficiency and cost-effectiveness using pre-pooling and enzymatic conversion
Source: BMC Res Notes. 2023 Jul 6;16:141. doi: 10.1186/s13104-023-06401-3 (PMC10326935; doi:10.1186/s13104-023-06401-3)
Supplement: Supplementary file 2 — Additional file 2: Text S2. EMCap protocol. [file 13104_2023_6401_MOESM2_ESM.docx]

**Additional file Text S2 (Hasegawa K *et al.*): The EMCap protocol (ver.20230120)**

**Protocol Overview**

**NOTE: The authors recommend that all steps of the experiment should be completed in one day.
If this is not possible, we recommend that Steps 4 to 8 be performed in one day.**

| **Step** | | **Manufacturer’ protocol(s) adopted** | **Reagents** |
| --- | --- | --- | --- |
| Step 1: | Preparation of DNA | - Covaris Quick Guide^1)^ | - 0.1× TE buffer (pH 8.0) |
| Step 2: | End repair, dA-tailing, adapter ligation, and clean-up of adaptor-ligated DNA | - NEBNext® Enzymatic Methyl-seq Kit (NEB #E7120S/L)^2)^ | - Water (Nacalai Tesque, 06442-95) - NEBNext Ultra II End Prep Reaction Buffer (NEB, E7120) - End Prep Enzyme Mix (NEB, E7120) - xGen Methyl UDI-UMI adapter (15 μM; IDT, 10006644; this product is no longer available) - NEBNext Ultra II Ligation Enhancer (NEB, E7120) - Ligation Master Mix (NEB, E7120) - NEBNext Sample Purification Beads (NEB, E7120) - Elution Buffer (NEB, E7120) - EtOH |
| Step 3: | Oxidation of 5-methylcytosines and 5-hydroxymethylcytosines by TET2 and clean-up of adaptor-ligated DNA without size selection | - NEBNext® Enzymatic Methyl-seq Kit (NEB #E7120S/L) ^2)^ | - TET2 Reaction Buffer Supplement (NEB, E7120) - Oxidation Supplement (NEB, E7120) - DTT (NEB, E7120) - Oxidation Enhancer (NEB, E7120) - TET2 (NEB, E7120) - Fe (II) solution (500 mM, NEB, E7120) - Stop Reagent (NEB, E7120) - NEBNext Sample Purification Beads (NEB, E7120) - Water (Nacalai Tesque, 06442-95) - EtOH |
| Step 4: | Sample pooling and hybridization | - SureSelectXT Low Input Target Enrichment System for Illumina Multiplexed Sequencing Platforms (Version F0, September 2022)^3)^ - xGen hybridization capture of DNA libraries For NGS target enrichment (Version 4)^4)^ | - Human Cot DNA (IDT, 1080577) - Water (Nacalai Tesque, 06442-95) - xGen Universal Blockers TS Mix (IDT, 1075474) - SureSelect RNase Block (Agilent Technologies, 5190-9686) - SureSelect Fast Hybridization Buffer (Agilent Technologies, 5190-9686) - SureSelect XT Human Methyl-Seq Capture Library (Agilent Technologies, 5190-4661) |
| Step 5: | Capture of DNA/RNA hybrids and washes | - SureSelectXT Low Input Target Enrichment System for Illumina Multiplexed Sequencing Platforms (Version F0, September 2022)^3)^ | - Dynabeads MyOne Streptavidin T1 magnetic beads (Thermo Fisher Scientific, 65601) - SureSelect Binding Buffer (Agilent Technologies, 5190-9687) - SureSelect Wash Buffer 1 (Agilent Technologies, 5190-9687) - SureSelect Wash Buffer 2 (Agilent Technologies, 5190-9687) - 0.1 N NaOH |
| Step 6: | Deamination of cytosines using APOBEC and clean-up of deaminated DNA | - NEBNext® Enzymatic Methyl-seq Kit (NEB #E7120S/L) ^2)^ | - Water (Nacalai Tesque, 06442-95) - APOBEC Reaction Buffer (NEB, E7120) - BSA (NEB, E7120) - APOBEC (NEB, E7120) - NEBNext Sample Purification Beads (NEB, E7120) - Elution Buffer (NEB, E7120) - EtOH |
| Step 7: | PCR amplification, clean-up of amplified library, and quantification of post-capture library | - NEBNext® Enzymatic Methyl-seq Kit (NEB #E7120S/L) ^2)^ | - PCR primers (P5) - PCR primers (P7) - NEBNext Q5U Master Mix (NEB, E7120) - NEBNext Sample Purification Beads (NEB, E7120) - Elution Buffer (NEB, E7120) - EtOH |
| Step 8: | Library quantification | - | - |

| **Underlines indicate modifications from the manufacturer’s protocols** | |
| --- | --- |
| **Step 1** | **Preparation of DNA** |
| **1.1** | **Shearing DNA** |
| **1.1.1** | Genomic DNA (gDNA) samples (200 ng) dissolved in 50 μL of 0.1× TE buffer (pH 8.0) are sheared with the Covaris S220 system (Covaris) to a peak top size of 300 bp.  **Note: Covaris conditions below comprise our laboratorial protocol. Adopt the protocol at each laboratory and consider checking the size distributions using a Bioanalyzer or TapeStation after shearing the DNA.**   \| **Covaris conditions for 300 bp** \| \| \| --- \| --- \| \| **Duty Factor (%)** \| 10 \| \| **Peak Incident Power (W)** \| 140 \| \| **Cycles per Burst** \| 200 \| \| **Duration (s)** \| 90 \| \| **Temperature (℃)** \| 7 \| \| **Water Level** \| 12 \| |
| **1.1.2** | Transfer the 50 μL of sheared DNA to a new PCR tube for End Prep. |
|  |  |
| **Step 2** | **End repair, dA-tailing, adapter ligation, and clean-up of adaptor-ligated DNA** |
| **2.1** | **End repair and dA-tailing** |
| **2.1.1** | On ice, mix the following components in a sterile nuclease-free PCR tube:   \| **COMPONENT** \| **VOLUME** \| \| \| --- \| --- \| --- \| \| Fragmented DNA (200 ng) \| 50 \| μL \| \| NEBNext Ultra II End Prep Reaction Buffer \| 7 \| μL \| \| NEBNext Ultra II End Prep Enzyme Mix \| 3 \| μL \| \| **Total volume** \| **60** \| **μL** \|   **Note: If the volume of the fragmented DNA solution is less than 50 μL, add DW to adjust it to 50 μL.** |
| **2.1.2** | Pipette the entire volume up and down at least 10 times to mix it thoroughly. |
| **2.1.3** | Place it in a thermocycler, and run the following program:  (the heated lid is set to 75°C)   \| **Temperature** \| **Time** \| \| --- \| --- \| \| 20℃ \| 30 min \| \| 65℃ \| 30 min \| \| 4℃ \| ∞ \| |
| **2.2** | **Adaptor ligation** |
| **2.2.1** | Add 2.5 μL of xGen Methyl UDI-UMI Adapter (15 μM) and mix 10 times by pipetting (total: 62.5 μL).  **Caution 1: The xGen Methyl UDI-UMI Adapter must be added before the following mixture (2.2.2). Caution 2: The xGen Methyl UDI-UMI Adapter must not be mixed with the following mixtures (2.2.2).** |
| **2.2.2** | On ice, add the following components directly to the 62.5 μL reaction mixture and mix well:   \| **COMPONENT** \| **VOLUME** \| \| \| --- \| --- \| --- \| \| Fragmented DNA (200 ng) \| 62.5 \| μL \| \| NEBNext Ultra II Ligation Master Mix \| 30 \| μL \| \| NEBNext Ligation Enhancer \| 1 \| μL \| \| **Total volume** \| **93.5** \| **μL** \| |
| **2.2.3** | Place it in a thermocycler, and run the following program:  (the heated lid is set to OFF)   \| **Temperature** \| **Time** \| \| --- \| --- \| \| 20℃ \| 60 min \| \| 4℃ \| ∞ \| |
|  | **Safe stopping point: samples can be stored overnight at −20°C.** |
| **2.3** | **Clean-up of adaptor-ligated DNA without size selection** |
| **2.3.1** | Vortex NEBNext Sample Purification Beads to resuspend them. |
| **2.3.2** | Add 110 μL (1.2×) of the resuspended NEBNext Sample Purification Beads to each sample. Mix well by pipetting up and down at least 10 times. |
| **2.3.3** | Incubate samples on a bench top for 5 min at room temperature. |
| **2.3.4** | Place the tubes against an appropriate magnetic stand to separate the beads from the supernatant. |
| **2.3.5** | After 5 min, carefully remove and discard the supernatant. Be careful not to disturb the beads that contain DNA targets.  **Caution: Do not discard the beads.** |
| **2.3.6** | Add 200 μL of 80% freshly prepared ethanol to the tubes while in the magnetic stand. Incubate at room temperature for 30 s, and then, carefully remove and discard the supernatant. Be careful not to disturb the beads that contain DNA targets. |
| **2.3.7** | Repeat the ethanol wash once for a total of two washes. Be sure to remove all visible liquid after the second wash using a p10 pipette tip. |
| **2.3.8** | Air dry the beads for 2 min while the tubes are on the magnetic stand with the lid open.  **Caution: Do not over-dry the beads.** |
| **2.3.9** | Remove the tubes from the magnetic stand. Elute the DNA target from the beads by adding 28 μL of Elution Buffer. |
| **2.3.10** | Mix well by pipetting up and down 10 times. Incubate it for 2 min at room temperature. If necessary, quickly spin the sample to collect the liquid from the sides of the tube before placing it back on the magnetic stand. |
| **2.3.11** | Place the tube on the magnetic stand. After 3 min, transfer 28 μL of the supernatant to a new PCR tube. |
|  | **Safe stopping point: samples can be stored overnight at −20°C.** |
| **Step 3** | **Oxidation of 5-methylcytosines and 5-hydroxymethylcytosines by TET2 and clean-up of adaptor-ligated DNA without size selection** |
| **3.1** | **Oxidation of 5-methylcytosines and 5-hydroxymethylcytosines** |
| **3.1.1** | Prepare TET2 Buffer. Use option A if you have NEB (E7120S) and option B if you have NEB (E7120L).  **Note: The TET2 Reaction Buffer Supplement is a powder. Centrifuge it before use to ensure that it is at the bottom of the tube.**  3.1.1 A: Add 100 μL of TET2 Reaction Buffer to one tube of the TET2 Reaction Buffer Supplement and mix it well.  3.1.1B: Add 400 μL of TET2 Reaction Buffer to one tube of the TET2 Reaction Buffer Supplement and mix it well. |
| **3.1.2** | On ice, add the following components directly to the 28 μL of adapter-ligated DNA (2.3.11).   \| **COMPONENT** \| **VOLUME** \| \| \| --- \| --- \| --- \| \| Adaptor-ligated DNA \| 28 \| μL \| \| TET2 Reaction Buffer (3.1.1) \| 10 \| μL \| \| Oxidation Supplement \| 1 \| μL \| \| DTT \| 1 \| μL \| \| Oxidation Enhancer \| 1 \| μL \| \| TET2 \| 4 \| μL \| \| **Total volume** \| **45** \| **μL** \| |
| **3.1.3** | Mix thoroughly by vortexing, and centrifuge briefly. For multiple reactions, a master mix of the reaction components can be prepared before addition to the sample DNA. |
| **3.1.4** | Dilute the 500 mM Fe(II) solution by adding 1 μL to 1249 μL of water.  **Note: Use the solution immediately. Do not store it. Discard it after use.** |
| **3.1.5** | Combine Diluted Fe(II) Solution and EM-seq DNA with oxidation enzymes   \| **COMPONENT** \| **VOLUME** \| \| \| --- \| --- \| --- \| \| EM-seq DNA \| 45 \| μL \| \| Diluted Fe(II) Solution (3.1.4) \| 5 \| μL \| \| **Total volume** \| **50** \| **μL** \| |
| **3.1.6** | Mix thoroughly by vortexing or by pipetting up and down at least 10 times, and centrifuge briefly. |
| **3.1.7** | Incubate it at 37°C for 1 h in a thermocycler.  (the heated lid is set to 50°C)   \| **Temperature** \| **Time** \| \| --- \| --- \| \| 37℃ \| 60 min \| |

| **3.1.8** | Transfer the samples to ice and add 1 μL of Stop Reagent.   \| **COMPONENT** \| **VOLUME** \| \| \| --- \| --- \| --- \| \| EM-seq DNA \| 50 \| μL \| \| Stop Reagent \| 1 \| μL \| \| **Total volume** \| **51** \| **μL** \| |
| --- | --- | --- | --- | --- | --- | --- | --- | --- | --- | --- | --- | --- | --- |
| **3.1.9** | Mix thoroughly by vortexing or by pipetting up and down at least 10 times, and centrifuge briefly. |
| **3.1.10** | Incubate it at 37°C for 30 min then at 4°C in a thermocycler.  (the heated lid is set to 50°C)   \| **Temperature** \| **Time** \| \| --- \| --- \| \| 37℃ \| 30 min \| \| 4℃ \| ∞ \| |
|  | **Safe stopping point: samples can be stored overnight at either 4°C in the thermocycler**  **or at −20°C in the freezer.** |
| **3.2** | **Clean-up of adaptor-ligated DNA without size selection** |
| **3.2.1** | Vortex the NEBNext Sample Purification Beads to resuspend them. |
| **3.2.2** | Add 90 μL (1.8×) of resuspended NEBNext Sample Purification Beads to each sample. Mix well by pipetting up and down at least 10 times. |
| **3.2.3** | Incubate the samples on a bench top for 5 min at room temperature. |
| **3.2.4** | Place the tubes against an appropriate magnetic stand to separate the beads from the supernatant. |
| **3.2.5** | After 5 min, carefully remove and discard the supernatant. Be careful not to disturb the beads that contain DNA targets.  **Caution: Do not discard the beads.** |
| **3.2.6** | Add 200 μL of 80% freshly prepared ethanol to the tubes while in the magnetic stand. Incubate them at room temperature for 30 s, and then, carefully remove and discard the supernatant. Be careful not to disturb the beads that contain DNA targets. |
| **3.2.7** | Repeat the ethanol wash once for a total of two washes. Be sure to remove all visible liquid after the second wash using a p10 pipette tip. |
| **3.2.8** | Air dry the beads for 2 min while the tubes are on the magnetic stand with the lid open.  **Caution: Do not over-dry the beads.** |
| **3.2.9** | Remove the tubes from the magnetic stand. Elute the DNA target from the beads by adding 12 μL of water. |
| **3.2.10** | Mix well by pipetting up and down 10 times. Incubate it for 2 min at room temperature. If necessary, quickly spin the sample to collect the liquid from the sides of the tube before placing it back on the magnetic stand. |
| **3.2.11** | Place the tube on the magnetic stand. After 3 min, transfer 12 μL of the supernatant to a new PCR tube. |
|  | **Safe stopping point: samples can be stored overnight at −20°C.** |

| **Step 4** | **Sample pooling and hybridization** |
| --- | --- |
| **4.1** | **Sample pooling** |
| **4.1.1** | Pool 12 μL each of the adapter-ligated and TET2-treated DNA samples.  **Caution: The number of pools here is limited to four samples.** |
| **4.2** | **Hybridization** |
| **4.2.1** | Add 5 μL of Human Cot DNA to the pooled DNA. |
| **4.2.2** | The pooled DNA (4.2.1) is then dried using a centrifugal concentrator for 45 min. |
| **4.2.3** | The dried DNA is resuspended in 12 μL of water. |
| **4.2.4** | Add 1.25 μL of xGen Universal Blockers TS Mix to the resuspended DNA (13.25 μL in total). |
| **4.2.5** | Mix thoroughly by vortexing for 5 s, and centrifuge briefly. |
| **4.2.6** | Incubate the DNA solution in a thermocycler.  (the heated lid is set to 105°C)   \|  \| **Temperature** \| **Time** \| **Number of cycles** \| \| --- \| --- \| --- \| --- \| \| Step 1 (see 4.2.7) \| 95℃ \| 5 min \| 1 \| \| Step 2 (see 4.2.8) \| 65℃ \| 10 min \| 1 \| \| Step 3 (see 4.2.9) \| 65℃ \| 1 min \| 1 \| \| Step 4 \| 65℃ \| 1 min \| 60 \| \| 37℃ \| 3 s \| \| Step 5 \| 65℃ \| Hold \| 1 \| |
| **4.2.7** | During 4.2.6 Step 1, prepare 2 μL of 25% SureSelect RNase Block and keep it on ice.   \| **COMPONENT** \| **VOLUME** \| \| \| --- \| --- \| --- \| \| SureSelect RNase Block \| 0.5 \| μL \| \| Water \| 1.5 \| μL \| \| **Total volume** \| **2** \| **μL** \| |
| **4.2.8** | During 4.2.6 Step 2, prepare 13 μL of the probe hybridization mixture, and keep it at room temperature.   \| **COMPONENT** \| **VOLUME** \| \| \| --- \| --- \| --- \| \| 25% RNase Block solution (from step 4.2.7) \| 2 \| μL \| \| SureSelect XT Human Methyl-Seq Capture Library \| 5 \| μL \| \| SureSelect Fast Hybridization Buffer \| 6 \| μL \| \| **Total volume** \| **13** \| **μL** \| |
| **4.2.9** | During 4.2.6 Step 3, add 13 μL of probe hybridization mixture (4.2.8) to the DNA solution (13.25 μL) on a thermal cycler block. |
| **4.2.10** | Mix thoroughly by vortexing for 5 s, and centrifuge briefly.  **Caution: During 4.2.9 and 4.2.10, you can pause the thermocycler at Step3 only for a short time.** |
| **4.2.11** | Set the DNA solution back into the thermocycler immediately and proceed with the reaction (4.2.6 Step 4 and Step 5). |
|  |  |
| **Step 5** | **Capture and washing of DNA/RNA hybrids** |
| **5.1** | **Prepare streptavidin beads**  **Caution1: Dynabeads MyOne Streptavidin T1 magnetic beads must be kept at room temperature for at least 30 min.**  **Caution 2: This step starts 1 h after the end of the previous step.** |
| **5.1.1** | Vigorously resuspend the Dynabeads MyOne Streptavidin T1 magnetic beads using a vortex mixer. |
| **5.1.2** | Add 50 μL of the magnetic bead suspension to a 1.5 mL tube. |
| **5.1.3** | Add 200 μL of SureSelect Binding Buffer. |
| **5.1.4** | Mix the beads by pipetting up and down 10 times. |
| **5.1.5** | Place the tubes against an appropriate magnetic stand to separate the beads from the supernatant for 5 min. |
| **5.1.6** | Remove and discard the supernatant. |
| **5.1.7** | Repeat Step 5.1.3 through Step 5.1.6 for a total of three washes. |
| **5.1.8** | Transfer the resuspended beads (5.1.7) to a 0.2 mL tube of a new eight-tube strip. |
|  |  |
| **5.2** | **Capture hybrids using streptavidin beads, and elution of DNA** |
| **5.2.1** | After the hybridization program is complete, open the thermal cycler lid and directly transfer the volume of each hybridization reaction into a tube of resuspended beads (5.1.8). |
| **5.2.2** | Mix thoroughly by pipetting. |
| **5.2.3** | Mix for 30 min at room temperature using a plate mixer (1400–1800 rpm). |
| **5.2.4** | During the 30 min incubation, pre-warm 1400 μL of SureSelect Wash Buffer 2 at 70°C. |
| **5.2.5** | After the 30 min incubation, centrifuge the capture reaction tube briefly. |
| **5.2.6** | Place the tubes against an appropriate magnetic stand to separate the beads from the supernatant for 2 min. |
| **5.2.7** | Remove and discard the supernatant. |
| **5.2.8** | Add 200 μL of SureSelect Wash Buffer 1. |
| **5.2.9** | Mix thoroughly by pipetting up and down 20 times. |
| **5.2.10** | Place the tubes against an appropriate magnetic stand to separate the beads from the supernatant for 1 min. |
| **5.2.11** | Remove and discard the supernatant. |
| **5.2.12** | Add 200 μL of pre-warmed SureSelect Wash Buffer 2. |
| **5.2.13** | Mix the beads by pipetting up and down 15 times. |
| **5.2.14** | Mix thoroughly by vortexing and centrifuge briefly. |
| **5.2.15** | Incubate for 5 min at 70°C in a thermocycler. |
| **5.2.16** | Place the tubes against an appropriate magnetic stand to separate the beads from the supernatant for 1 min. |
| **5.2.17** | Remove and discard the supernatant. |
| **5.2.18** | Repeat Step 5.2.12 through Step 5.2.17 for a total of six washes. |
| **5.2.19** | Remove the residual buffer at the final wash procedure completely. |
| **5.2.20** | Add 4 μL of 0.1 N NaOH to the streptavidin beads directly in a 0.2 mL PCR tube. |
| **5.2.21** | Centrifuge briefly to spin it down, mix by vortexing, and centrifuge it briefly again.  **Caution: Be sure to mix by vortexing, since mixing by pipetting will result in beads remaining in the tip, leading to lower yields of final libraries.** |
| **5.2.22** | Place the tube on the magnetic stand. After 2 min, transfer 4 μL of the supernatant to a new PCR tube. |
|  |  |
| **Step 6** | **Deamination of cytosines using APOBEC and clean-up of deaminated DNA** |
| **6.1** | **Deamination of cytosines using APOBEC** |
| **6.1.1** | On ice, add the following components to the 4 μL of denatured DNA.   \| **COMPONENT** \| **VOLUME** \| \| \| --- \| --- \| --- \| \| Denatured DNA \| 4 \| μL \| \| Water \| 84 \| μL \| \| APOBEC Reaction Buffer \| 10 \| μL \| \| BSA \| 1 \| μL \| \| APOBEC \| 1 \|  \| \| **Total volume** \| **100** \| **μL** \| |
| **6.1.2** | Mix thoroughly by pipetting up and down 10 times, and centrifuge briefly. |
| **6.1.3** | Incubate at 37°C for 3 h and then at 4°C in a thermocycler.  (the heated lid is set to 105°C)   \| **Temperature** \| **Time** \| \| --- \| --- \| \| 37℃ \| 180 min \| \| 4℃ \| ∞ \| |
| **6.2** | **Clean-up of deaminated DNA** |
| **6.2.1** | Vortex the NEBNext Sample Purification Beads to resuspend them. |
| **6.2.2** | Add 100 μL (1×) of resuspended NEBNext Sample Purification Beads to each sample. Mix well by pipetting up and down at least 10 times. |
| **6.2.3** | Incubate the samples on a bench top for 5 min at room temperature. |
| **6.2.4** | Place the tubes against an appropriate magnetic stand to separate the beads from the supernatant. |
| **6.2.5** | After 5 min, carefully remove and discard the supernatant. Be careful not to disturb the beads that contain DNA targets.  **Caution: Do not discard the beads.** |
| **6.2.6** | Add 200 μL of 80% freshly prepared ethanol to the tubes while in the magnetic stand. Incubate them at room temperature for 30 s, and then, carefully remove and discard the supernatant. Be careful not to disturb the beads that contain DNA targets. |
| **6.2.7** | Repeat the ethanol wash once for a total of two washes. Be sure to remove all visible liquid after the second wash using a p10 pipette tip. |
| **6.2.8** | Air dry the beads for 90 s while the tubes are on the magnetic stand with the lid open.  **Caution: Do not over-dry the beads.** |
| **6.2.9** | Remove the tubes from the magnetic stand. Elute the DNA target from the beads by adding 20 μL of Elution Buffer. |
| **6.2.10** | Mix well by pipetting up and down 10 times. Incubate it for 1 min at room temperature. If necessary, quickly spin the sample to collect the liquid from the sides of the tube before placing it back on the magnetic stand. |
| **6.2.11** | Place the tube on the magnetic stand. After 3 min, transfer 20 μL of the supernatant to a new PCR tube. |
|  |  |
| **Step 7** | **PCR amplification, clean-up of amplified library, and quantification of post-capture library** |
| **7.1** | **PCR amplification** |
| **7.1.1** | On ice, add the following components to the eluted DNA.   \| **COMPONENT** \| **VOLUME** \| \| \| --- \| --- \| --- \| \| Eluted DNA \| 20 \| μL \| \| PCR primer (P5) \| 2.5 \| μL \| \| PCR primer (P7) \| 2.5 \| μL \| \| NEBNext Q5U Master Mix \| 25 \| μL \| \| **Total volume** \| **50** \| **μL** \| |
| **7.1.2** | Place it in a thermocycler, and run the following program:  (the heated lid is set to 105°C)   \|  \| **Temperature** \| **Time** \| **Number of cycles** \| \| --- \| --- \| --- \| --- \| \| Step 1 \| 98℃ \| 30 s \| 1 \| \| Step 2 \| 98℃ \| 10 s \| 9 \| \| 62℃ \| 30 s \| \| 65℃ \| 60 s \| \| Step 3 \| 65℃ \| 5 min \| 1 \| \| Step 4 \| 4℃ \| ∞ \|  \| |
| **7.2** | **Clean-up of amplified library** |
| **7.2.1** | Vortex the NEBNext Sample Purification Beads to resuspend them. |
| **7.2.2** | Add 45 μL (0.9×) of resuspended NEBNext Sample Purification Beads to each sample. Mix well by pipetting up and down at least 10 times. |
| **7.2.3** | Incubate the samples on a bench top for 5 min at room temperature. |
| **7.2.4** | Place the tubes against an appropriate magnetic stand to separate the beads from the supernatant. |
| **7.2.5** | After 5 min, carefully remove and discard the supernatant. Be careful not to disturb the beads that contain DNA targets.  **Caution: Do not discard the beads.** |
| **7.2.6** | Add 200 μL of 80% freshly prepared ethanol to the tubes while in the magnetic stand. Incubate it at room temperature for 30 s, and then, carefully remove and discard the supernatant. Be careful not to disturb the beads that contain DNA targets. |
| **7.2.7** | Repeat the ethanol wash once for a total of two washes. Be sure to remove all visible liquid after the second wash using a p10 pipette tip. |
| **7.2.8** | Air dry the beads for 2 min while the tubes are on the magnetic stand with the lid open.  **Caution: Do not over-dry the beads.** |
| **7.2.9** | Remove the tubes from the magnetic stand. Elute the DNA target from the beads by adding 21 μL of Elution Buffer. |
| **7.2.10** | Mix well by pipetting up and down 10 times. Incubate for 1 min at room temperature. If necessary, quickly spin the sample to collect the liquid from the sides of the tube before placing it back on the magnetic stand. |
| **7.2.11** | Place the tube on the magnetic stand. After 3 min, transfer 21 μL of the supernatant to 1.5 mL tube. |
|  |  |
| **Step 8** | **Library quantification** |
|  | Use a Bioanalyzer or TapeStation to determine the size distribution and concentration of the libraries. |

**Supplementary references:**

1. [**https://www.covaris.com/wp-content/uploads/pn_010308.pdf**](https://www.covaris.com/wp-content/uploads/pn_010308.pdf)
2. [**https://www.neb.com/-/media/nebus/files/manuals/manuale7120.pdf?rev=bfb32fbce7074e41bff0db9259b92c9c**](https://www.neb.com/-/media/nebus/files/manuals/manuale7120.pdf?rev=bfb32fbce7074e41bff0db9259b92c9c)
3. [**https://www.agilent.com/cs/library/usermanuals/public/G9703-90000.pdf**](https://www.agilent.com/cs/library/usermanuals/public/G9703-90000.pdf)
4. [**https://www.veritastk.co.jp/pdf/xgen-hybridization-capture-of-dna-libraries.pdf**](https://www.veritastk.co.jp/pdf/xgen-hybridization-capture-of-dna-libraries.pdf)
