# Supplementary material for: A capture methyl-seq protocol with improved efficiency and cost-effectiveness using pre-pooling and enzymatic conversion
Source: BMC Res Notes. 2023 Jul 6;16:141. doi: 10.1186/s13104-023-06401-3 (PMC10326935; doi:10.1186/s13104-023-06401-3)
Supplement: Supplementary file 10 — Additional file 10: Fig. S1. Comparisons of sequencing metrics among the Agilent’s SureSelect XT Human Methyl-Seq (BSCap) high, medium, low and EMCap. Fig. S2. Read depths and methylation values of individual target CpG sites with the minimum coverage of 1 in at least one of the eight libraries visualized for a 120 kb interval (hg19) from the HOXA gene cluster region on chromosome 7 using Integrative Genomics Viewer (IGV; https://igv.org/). [file 13104_2023_6401_MOESM10_ESM.pptx]

## Slide 1
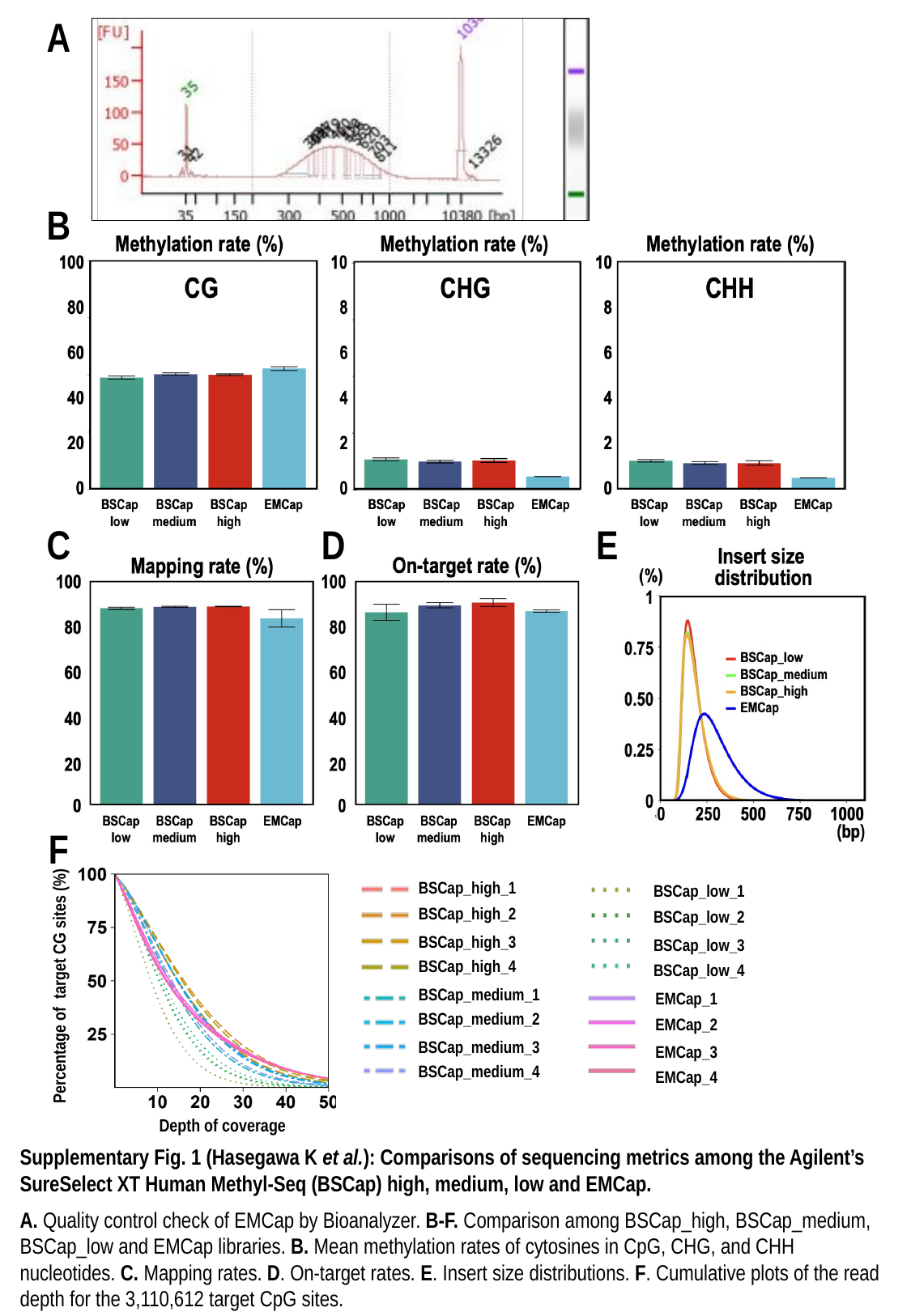

A
B
C
E
D
F
BSCap_high_1
BSCap_low_1
BSCap_high_2
BSCap_low_2
BSCap_high_3
BSCap_low_3
BSCap_high_4
BSCap_low_4
Percentage of target CG sites (%)
BSCap_medium_1
EMCap_1
BSCap_medium_2
EMCap_2
BSCap_medium_3
EMCap_3
BSCap_medium_4
EMCap_4
Depth of coverage
Supplementary Fig. 1 (Hasegawa K et al.): Comparisons of sequencing metrics among the Agilent’s SureSelect XT Human Methyl-Seq (BSCap) high, medium, low and EMCap.
A. Quality control check of EMCap by Bioanalyzer. B-F. Comparison among BSCap_high, BSCap_medium, BSCap_low and EMCap libraries. B. Mean methylation rates of cytosines in CpG, CHG, and CHH nucleotides. C. Mapping rates. D. On-target rates. E. Insert size distributions. F. Cumulative plots of the read depth for the 3,110,612 target CpG sites.

## Slide 2
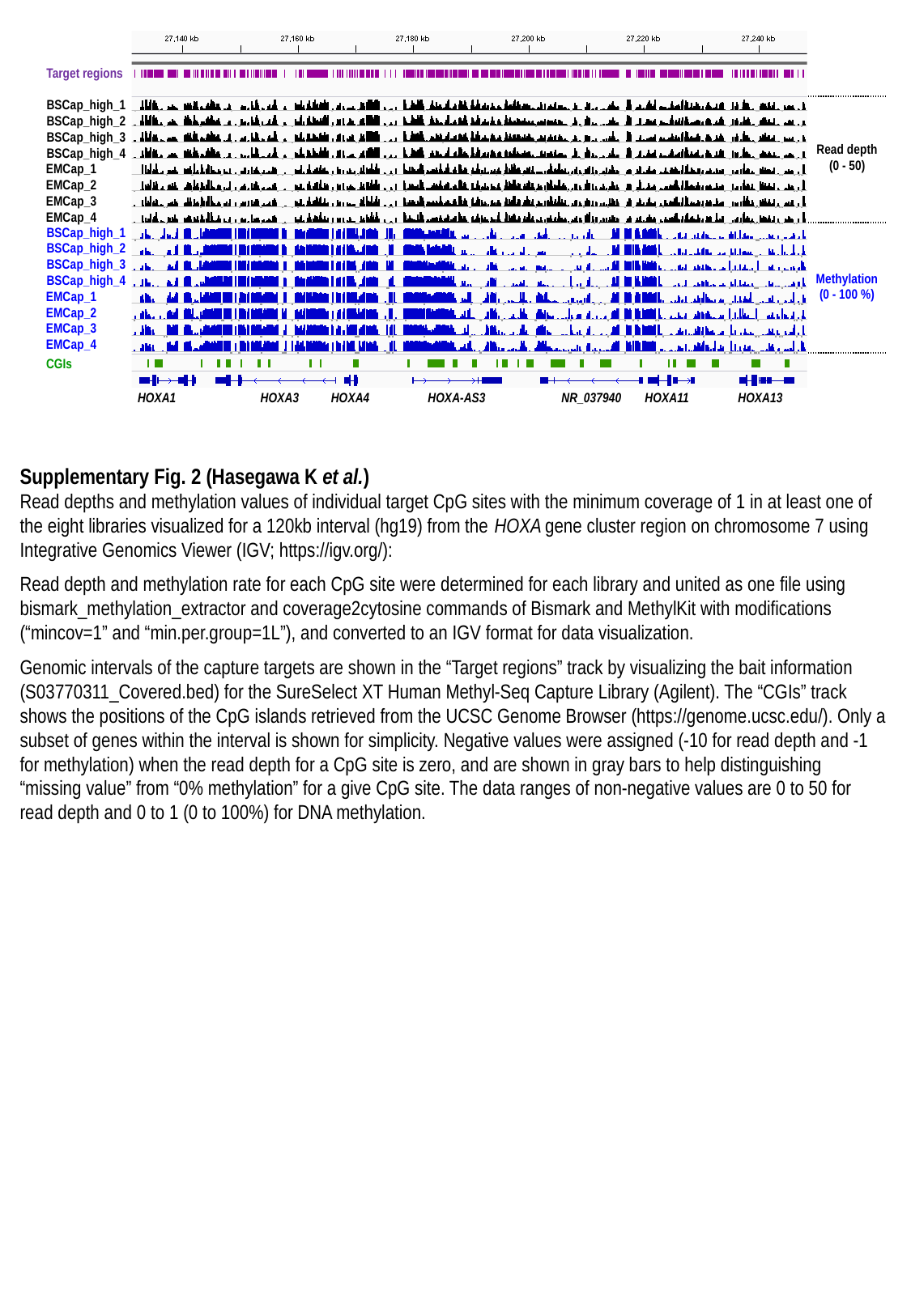

Target regions
BSCap_high_1
BSCap_high_2
BSCap_high_3
BSCap_high_4
EMCap_1
EMCap_2
EMCap_3
EMCap_4
Read depth
(0 - 50)
Methylation
(0 - 100 %)
BSCap_high_1
BSCap_high_2
BSCap_high_3
BSCap_high_4
EMCap_1
EMCap_2
EMCap_3
EMCap_4
CGIs
HOXA1
HOXA3
HOXA4
HOXA-AS3
NR_037940
HOXA11
HOXA13
Supplementary Fig. 2 (Hasegawa K et al.)
Read depths and methylation values of individual target CpG sites with the minimum coverage of 1 in at least one of the eight libraries visualized for a 120kb interval (hg19) from the HOXA gene cluster region on chromosome 7 using Integrative Genomics Viewer (IGV; https://igv.org/):
Read depth and methylation rate for each CpG site were determined for each library and united as one file using bismark_methylation_extractor and coverage2cytosine commands of Bismark and MethylKit with modifications (“mincov=1” and “min.per.group=1L”), and converted to an IGV format for data visualization.
Genomic intervals of the capture targets are shown in the “Target regions” track by visualizing the bait information (S03770311_Covered.bed) for the SureSelect XT Human Methyl-Seq Capture Library (Agilent). The “CGIs” track shows the positions of the CpG islands retrieved from the UCSC Genome Browser (https://genome.ucsc.edu/). Only a subset of genes within the interval is shown for simplicity. Negative values were assigned (-10 for read depth and -1 for methylation) when the read depth for a CpG site is zero, and are shown in gray bars to help distinguishing “missing value” from “0% methylation” for a give CpG site. The data ranges of non-negative values are 0 to 50 for read depth and 0 to 1 (0 to 100%) for DNA methylation.
